# 4367 Exploratory evaluation of an online educational intervention for JUUL use

**DOI:** 10.1017/cts.2020.128

**Published:** 2020-07-29

**Authors:** Eleanor L S Leavens, Matthew J. Carpenter, Tracy T. Smith, Nikki Nollen

**Affiliations:** 1 University of Kansas Frontiers

## Abstract

OBJECTIVES/GOALS: Initiation of JUUL use by young adults is one of the most significant issues of concern within the debate on vaping. Despite the proliferation of products and the surge in prevalence, no studies have investigated individual-level interventions or prevention strategies for pod-mod use. METHODS/STUDY POPULATION: Participants (*N* = 947) were young adults (<30 years old) recruited from Amazon’s Mechanical Turk based on smoking (never, former, and current smokers) and JUUL use status (never and current users), resulting in 6 use groups. In a pre-post design, participants completed baseline assessments, were presented with a brief JUUL-specific educational intervention, and completed post-assessment measures. The one-page intervention provided basic information about JUUL and stated that JUUL is harmful to non-smokers but could be beneficial to smokers if they completely switch. Primary outcomes were changes in JUUL knowledge, perceived harmfulness, intentions for future use, and motivation to change. RESULTS/ANTICIPATED RESULTS: Participants (*M_age_* = 26.1) were male (57%) and White (75%). Overall, the intervention increased JUUL-related knowledge, risk perceptions, commitment to quitting, and readiness to quit JUUL (*p*s<.01). Similarly, participants showed decreased interest in future JUUL use, interest in purchasing JUUL, and interest in future regular use (*p*s<.01). Non-JUUL users showed decreased interest in initiating JUUL use after viewing the intervention (*p*<.01). There were significant Time X Group interactions for JUUL-related knowledge (*p*<.001), with never JUUL/never smokers showing the greatest increase in product knowledge following the intervention. However, no other interaction effects were significant. DISCUSSION/SIGNIFICANCE OF IMPACT: The intervention was effective in increasing knowledge and risk perceptions while reducing intentions for future use. The intervention was most effective in increasing knowledge among non-users, suggesting that brief educational interventions may be useful tools for preventing pod-mod initiation. CONFLICT OF INTEREST DESCRIPTION: Dr. Carpenter has received consulting honoraria from Pfizer. All other authors have no conflicts to disclose.

